# Prognostic significance of *K-Ras* mutation rate in metastatic colorectal cancer patients

**DOI:** 10.18632/oncotarget.5231

**Published:** 2015-08-19

**Authors:** Bruno Vincenzi, Chiara Cremolini, Andrea Sartore-Bianchi, Antonio Russo, Francesco Mannavola, Giuseppe Perrone, Francesco Pantano, Fotios Loupakis, Daniele Rossini, Elena Ongaro, Erica Bonazzina, Emanuela Dell'Aquila, Marco Imperatori, Alice Zoccoli, Giuseppe Bronte, Giovanna De Maglio, Gabriella Fontanini, Clara Natoli, Alfredo Falcone, Daniele Santini, Andrea Onetti-Muda, Salvatore Siena, Giuseppe Tonini, Giuseppe Aprile

**Affiliations:** ^1^ Department of Medical Oncology, Campus Bio-Medico University of Rome, Rome, Italy; ^2^ Azienda Ospedaliero-Universitaria Pisana, Istituto Toscano Tumori, Pisa, Italy; ^3^ Niguarda Cancer Center, Ospedale Niguarda Ca' Granda, Milano, Italy; ^4^ Department of Surgical, Oncological and Oral Sciences, Section of Medical Oncology, University of Palermo, Palermo, Italy; ^5^ Università Cattolica del Sacro Cuore, Rome, Italy; ^6^ Department of Anatomical Pathology, Campus Bio-Medico University of Rome, Rome, Italy; ^7^ Department of Medical Oncology, Azienda Ospedaliero-Universitaria, Udine, Italy; ^8^ Dipartimento di Patologia Chirurgica, Medica, Molecolare e dell'Area Critica, Università di Pisa, Pisa, Italy; ^9^ Department of Medical, Oral and Biotechnological Sciences, University “G. D'Annunzio”, Chieti, Italy

**Keywords:** K-Ras, mutation rate, colorectal cancer, bevacizumab, prognosis

## Abstract

Introduction: Activating mutations of K-Ras gene have a well-established role as predictors of resistance to anti-EGFR monoclonal antibodies in metastatic colorectal cancer (mCRC) patients. Their prognostic value is controversial, and no data regarding the prognostic value of mutation rate, defined as the percentage of mutated alleles/tumor sample, are available. We aimed to evaluate the prognostic value of K-Rasmutation rate in a homogenous cohort of mCRC patients receiving first-line doublet plus bevacizumab.

Patients and Methods: This retrospective study enrolled 397 K-Ras mutant mCRC patients from 6 Italian centers, and 263 patients were fully evaluable for our analysis. K-Ras mutation rate was assessed by pyrosequencing. Patients with less than 60% of cancer cells in tumor tissue were excluded. No patients received anti-EGFR containing anticancer therapy, at any time. Median mutation rate was 40% and was adopted as cut-off. The primary and secondary endpoints were PFS and OS respectively.

Results: At univariate analysis, K-Ras mutation rate higher than 40% was significantly associated with lower PFS (7.3 *vs* 9.1 months; *P* < 0.0001) and OS (21 *vs* 31 months; *P* = 0.004). A multivariate model adjusted for age at diagnosis, site of origin of tumor tissue (primary *vs* metastases), referral center, number of metastatic sites, and first-line chemotherapy backbone, showed that K-Ras mutation rate remained a significant predictor of PFS and OS in the whole population.

Discussion: Our data demonstrate an association between K-Ras mutation rate and prognosis in mCRC patients treated with bevacizumab-containing first-line therapy. These data deserve to be verified in an independent validation set.

## INTRODUCTION

The Kirsten Rat Sarcoma (K-Ras) oncogene encodes for a 189 amino acids protein, also called p21, a member of RAS superfamily proteins [1]. Mutations in K-Ras gene are found in approximately 30% of all human cancers [2] and in 35-45% of colorectal cancers (CRCs) [3–5]. The 95% of K-Ras mutations occurs in codons 12 and 13 while mutations in codons 61, 146 and other are less frequent in CRC, accounting for 5% of all mutations [6]. Activating mutations of the K-Ras oncogene is an established predictive biomarker for resistance to anti-epidermal growth factor receptor (anti-EGFR) therapies in metastatic CRC (mCRC). Even though selection of Ras wild type (i.e. K-Ras and N-Ras exons 2, 3 and 4) greatly improve efficacy of EGFR-targeted agents by excluding 50% to 60% of patients with tumors refractory to EGFR blockade [7–8], molecular alterations in additional nodes of the EGFR signalling network also seem to be clinically relevant. Among these, downstream mutations such as in B-Raf and PIK3CA genes have been investigated through several sequencing methods [9–11].

Despite of the well known importance of K-Ras mutations as predictive factor to anti-EGFR drugs administration, there is no consensus about the prognostic value of mutational status of K-Ras in CRC patients. Several reports have indicated that K-Ras mutations are negative prognostic markers and portend a poorer outcome in CRC [12] and a meta-analysis of 3439 patients reported that mutation on codon 12 glycine to valine has a statistically significant impact on failure-free survival (FFS) (*P* = 0.004, HR:1.3) and overall survival (OS) (*P* = 0.008, HR:1.29) and it is associated with a more aggressive biological behaviour of tumor [13]. A retrospective analysis of FOCUS trial showed that activating mutation in K-Ras and B-Raf oncogene are associated with shorter survival: patients harbouring K-Ras mutation have worse OS than patients with K-Ras wt tumors (HR:1.24; 95%CI:1,06-1.46; *P* = 0.008), but there is no evidence of an effect on progression free survival (PFS) (HR:1.14; 95%CI:0,98-1.33; *P* = 0.09) [14]. A recent post hoc analysis of the TRIBE study confirmed the negative prognostic role of K-Ras mutation in patients with mCRC treated with bevacizumab; more in details: the OS of all wt Ras patients was 34.4 months and 41.7 months when they received bevacizumab and FOLFIRI or bevacizumab and FOLFOXIRI respectively (HR: 0.84, 95%CI: 0.51-1.38), while the OS of mutant Ras patients was 23.1 months and 28.6 months when they received bevacizumab and FOLFIRI or bevacizumab and FOLFOXIRI [15] respectively (HR: 0.86, 95%CI: 0.61 to 1.23). The PFS of all Ras wt patients was 11.3 months *vs* 13.3 months in Ras mutant patients treated with bevacizumab and FOLFIRI or bevacizumab and FOLFOXIRI.

Up to now different methods for K-Ras mutation analysis have been used in experimental settings or in clinical trials in order to transfer useful predictive and prognostic information to clinical practice. Currently, the use of more sensitive methods has significantly improved the detection of K-Ras mutations. Interestingly while K-Ras mutation status has been always considered a dichotomic information, the recently use of technologies such as pyrosequincing, allow to identify an additional parameter i.e. the mutation rate, that is a measure of the rate of mutant alleles. The introduction and understanding of the biological significance and potential clinical impact of this new parameter could represent a high priority for patients evaluation. Indeed to our knowledge, there are no data about prognostic and\or predictive significance of rate of mutation of K-Ras in tumor specimens of mCRC patients treated with bevacizumab-containing chemotherapy in order to identify a specific subgroup of mutant K-Ras patients receiving a significant prognostic benefit from adding bevacizumab. We aimed to evaluate the correlation between rate of mutation of K-Ras and response to bevacizumab-containing treatment as well as its prognostic effect.

## RESULTS

### Patients' population

**Figure 1:**
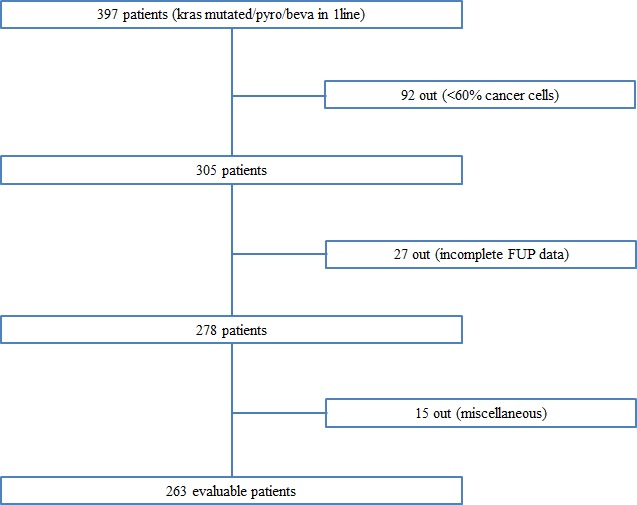
Diagram of the patients' cohort enrolled in the study

In this study we recruited 397 patients; 92 patients were excluded because of insufficient percentage of tumor cells in tissue samples (< 60%) and other 42 patients because of incomplete follow up or other missing data. We totally analyzed a population of 263 patients, 144 (54.6%) male and 119 (43.4%) female. The Figure 1 provides an overview diagram indicating the number of patients enrolled and analyzed in this study (Figure 1). K-Ras determination was performed on primary tumors for 69 (26.2%) patients and on metastatic cancer sites for 194 (73.8%). The results showed that mutant codon 12 was present in 211 (80%) patients, mutant codon 13 in 44 (17%) patients, while other rare mutations of K-Ras were present in 8 (3%) patients. All enrolled subjects have available data about bevacizumab-chemotherapy regimens, in particular all patients received bevacizumab; 201 (76.4%) patients received a first-line containing irinotecan, while 62 (23.6%) an oxaliplatin containing schedule. Among the whole population 191 (72.6%) of patients experienced a second-line therapy and 122 (46.4%) a third line. The majority of patients who showed one organ compromised were 178 (67.7%), multi-organ involvement refer to 49 (18.6%) patients with two organs implicated, while 36 (13.7%) subjects presented 3 or more interested organs. Objective tumor response (achieved as a confirmed PR or CR) was observed in 49% of the whole population. The median PFS and OS of mutant K-Ras patients were 8.25 months (95%CI: 7.6-8.9) and 26 months (95%CI: 22.03-29.97) respectively. Characteristics of evaluable patients were summarised in Table 1.

**Table 1:** Patients' features

	Absolute number	%
Number of evaluable patients	263	100
Available data about Bevacizumab-chemotherapy	263	100
Gender (male/female)	144/119	54.6/43.4
K-Ras determination (primary vs met)	194/69	73.8/26.2
Second Line	191	72.6
Third Line	122	46.4
Irinotecan based/Oxaliplatin based	201/62	76.4/23.6
Number of involved organs 1 2 3 or more	178 49 36	67.7 18.6 13.7
Cumulative response rate (cPR+cCR)	49%	-
PFS	8.25 months	7.6-8.9
OS	26 months	22-29.9

### Prognostic value of mutant K-Ras alleles: univariate analysis

**Figure 2:**
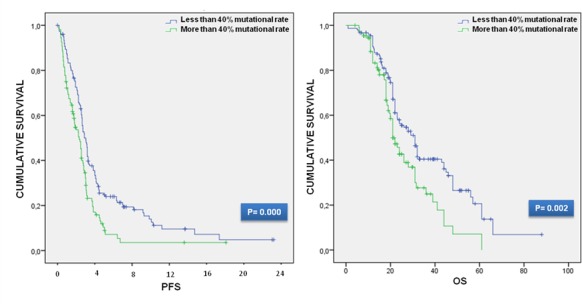
Kaplan-Meier curves for PFS and OS

In order to investigate the prognostic value of K-Ras mutation rate we firstly performed a survival analysis for the calculation of PFS and OS. We divided subjects into three groups: those mutated in codon 12, 13 and those with mutation on K-Ras rare codons. Each group was then dichotomized for the presence of a mutation rate higher or lower than 40%; the total number of the analyzed patients with a mutation rate > 40% was 109, while that of patients with a mutation rate < 40% was 154. The survival data were then used for the univariate analysis using the log rank test. We obtain that higher mutation rate resulted in a poor outcome in terms of PFS and OS. In particular the median PFS for mutant K-Ras patients was 8.25 months (95%CI: 7.48-9.01), but the PFS in mutant codon 12 patients with < 40% of mutation rate was 9.45 months (95%CI: 8.47-10.42) and that with ≥ 40% was 7.5 months (95%CI: 5.78-9.21). The median PFS in mutant codon 13 K-Ras patients was 8.9 months (95%CI: 7.69-10.10), the median PFS in mutant codon 13 K-Ras patients with < 40% and ≥ 40% was 10.25 months (95%CI: 8.15-12.34) and 7.25 months (95%CI: 14.40-43.59) respectively. The median PFS for other K-Ras mutations was 5.5 months (95%CI: 2.95-8.04), while the PFS for < 40% and ≥ 40% patients was 7.25 months (95%CI: 2.44-12.05) and 5.5 months (95%CI: 2.68-7.76) in that order. Regarding the median survival, OS of mutant K-Ras patients treated with a first line bevacizumab-containing treatment was 26 months (95%CI: 22.03-29.97). The median OS of mutant codon 12 patients was 28 months (95%CI: 23.67-32.32), but the OS in mutant codon 12 patients with < 40% was 31 months (95%CI: 27.00-34.99), while the OS in mCRC patients with codon 12 K-Ras mutation ≥ 40% was 23 months (95%CI: 19.79-26.20). The median OS in mutant codon 13 patients was 21 months (95%CI: 16.63-25.36); the OS in mutant codon 13 K-Ras patients with < 40% and ≥ 40% was 24 months (95%CI: 19.41-28.58) and 17 months (95%CI: 11.61-22.78) respectively. The OS of other rare mutant codons K-Ras patients was 31 months. In the univariate survival analysis, the patients with a mutation rate higher than 40% was significantly associated with both PFS (log-rank *P* = 0.000) and OS (log-rank *P* = 0.002) with PFS and OS median value indicative of worse survival (median PFS: 7.25 months (95%CI: 5.76-8.73) vs 9 months (95%CI: 7.96-10.03) and median OS: 21 months (95%CI: 18.35-23.64) vs 31 months (95%CI: 26.66-35.33)) correspondingly. The different subgroups survival data and Kaplan Meier curves are shown in Table 2 and Figure 2.

A bioinformatic analysis was also conducted on a separate cohort consisting of 20 patients radically resected for liver metastasis and treated with chemotherapy. Taking advantage of the availability of primary and matched liver metastasis samples, we performed a correlation analysis finding a positive association (*P* = 0.030) between K-Ras mutation rate on the two sites. A subsequent Kaplan Meier survival analysis did not find any ability of K-Ras mutation allele frequency in predicting OS (*P* = 0.88), but we found that the cut-off (35%) generated through ROC method was very close to the one (40%) we used to dichotomize our cohort.

**Table 2:** Comparisons table and log-rank test for prognostic factor categories

POPULATION [K-Ras mutated codons]	PFS (95%CI) [mts]	OS (95%CI) [mts]
Codon 12, all patients (pts)	8.25 (95%CI: 7.48-9.01)	28 (95%CI: 23.67-32.32)
Codon 12, pts with >40% vs ≤40% mutant alleles	7.45 (95%CI: 5.78-9.21) vs 9.45 (95%CI: 8.47-10.4)	23 (95%CI: 19.79-26.20) vs 31 (95%CI: 27.00-34.99)
Codon 13, all pts	8.90 (95%CI: 7.69-10.10)	21 (95%CI: 16.63-25.36)
Codon 13, pts with >40% vs ≤40% mutant alleles	7.25 (95%CI: 3.6-10.89) vs 10.25 (95%CI: 8.15-12.32)	17 (95%CI: 11.61- 22.78) vs 24 (95%CI: 19.41- 28.58)
Rare codons, all pts	5.50 (95%CI: 2.95- 8.04)	31 (95%CI: 27.03-34.03)
Rare codons, pts with >40% vs ≤40% mutant alleles	5.5 (95%CI: 10.75-31.04) vs 7.25 (95%CI: 2.44-12.05)	-
All K- Ras mutated codons, pts with >40% vs ≤40% mutant alleles	7.25 (95%CI: 5.76-8.73) vs 9 (95%CI: 7.96-10.03)	21 (95%CI: 18.35-23.64) vs 31 (95%CI: 26.66-35.33)
Log-Rank P	P=0.000	P=0.002

### Prognostic value of mutant K-Ras alleles: multivariate analysis

Since we have shown that the presence of more than 40% of mutation rate were associated with worse PFS and OS, we also applied a Cox's regression model with these factors included in order to estimate the independent effect of the mutation rate on the two outcomes. This unadjusted analysis provide the following results: for PFS an HR = 0.63 (95%CI: 0.46-0.80) with a *P* < 0.001, and for OS an HR = 0.66 (95%CI: 0.44-0.84) with a *P* = 0.004. Moreover the multivariate Cox proportional hazards analysis, performed to compare prognostic factors of survival after adjustment for the impact of other factors (age at diagnosis, tumor tissue used for molecular analysis, reference center, number of metastatic sites, first-line chemotherapy) displayed a significative association between mutation rate and the two outcome' predictors (for PFS: HR: 0.67, 95%CI: 0.53-0.82, *P* = 0.003; for OS: HR: 0.67, 95%CI: 0.53-0.82, *P* = 0.003). Finally, we investigate the independent effect of mutation rate (more or less than 40% mutant alleles) of codons 12, 13 and rare codons introducing them into the multivariate analysis; among these only codon' 12 mutation rate present a significant association with PFS (HR: 0.71, 95%CI: 0.52-0.85, *P* = 0.003) and OS (HR: 0.71, 95%CI: 0.52-0.85, *P* = 0.003).

Overall, these results demonstrated that K-Ras alleles mutation rate remained a significant predictor of PFS and OS (codon 13 and rare codons did not reach statistical significance probably due to the low number of patients) (Table 3).

**Table 3:** Multivariate Cox proportional hazards regression analysis

	PFS	OS
Survival Analysis (unadjusted)	HR: 0.63 (0.46-0.80) P < 0.001	HR: 0.66 (0.44-0.84) P= 0.004
Survival Analysis (adjusted)	HR: 0.67 (0.53-0.82) P= 0.003	HR: 0.71 (0.52-0.85) P= 0.012
Codon 12	HR: 0.71 (0.52-0.85) P= 0.003	HR: 0.71 (0.52-0.85) P= 0.011
Codon 13	HR: 0.62 (0.25-1.04) P= 0.066	HR: 0.579 (0.26-1.28) P= 0.179
Rare codons	HR: 0.19 (0.02-2.08) P= 0.397	HR: 0.43 (0.06-3.05) P= 0.397

## DISCUSSION

In the era of personalized medicine, the improved understanding of the EGFR pathway clarified that mutations of Ras genes are established negative predictive factors with regard to the use of anti-EGFR monoclonal antibodies. Not only the negative predictive value of K-Ras exon 2 mutations was validated in a number of studies [3, 5], but also the analysis of other mutations such as codon 61 in exon 3 and codon 146 in exon 4 may predict resistance to these drugs [16]. Moreover, since tumors harbouring any Ras mutation do not respond to cetuximab or panitumumab [17], an expanded Ras analysis, including N-Ras exon 2, 3 and 4, has been incorporated in the baseline evaluation of mCRC patients before treatment selection and the use of anti-EGFR monoclonal antibodies has been restricted to patients with Ras wild-type tumors.

Oppositely, the influence of K-Ras mutations on overall patients' outcome is much less evident, indeed its prognostic value for patients with colorectal cancer is still debated. Even if K-Ras mutations seem not to correlate with the prognosis of patients with colorectal cancer the association remains to be confirmed with a more precise analysis of a large sample [18].

One of the reasons of these inconclusive results can be also attributed to cancer heterogeneity. Many tumors present heterogeneity within their cell population. This lack of cellular homogeneity may arise because of differential nutrient status due to an altered cancer induced microcirculations, normal cells' infiltration into the tumor mass and to the hierarchical natures of the cell populations from which cancers arise. Recent findings demonstrated the complexity of settle and understand the heterogeneity of malignant disease, especially in an advanced setting. Some authors suggested that the molecular profile of the primary tumor might significantly differ from that of a corresponding metastatic site and might not reflect molecular aberrations accumulated as a consequence of selection pressure caused by applied cancer therapies. In addition, the molecular profile(s) of different metastatic sites might be disparate [19–21]. The molecular discordances between anatomic site locations of the same patient may hence reflect cancer heterogeneity [22]. Despite these data, other scientific reports established that, especially in patients never exposed to anti-EGFRs, the rate of concordance between primary tumor and corresponding metastatic sites is very high [23–25], and our data are in accordance with those findings. Moreover in the cancers' genetic heterogeneity scenario is important to take into account that the percentage of K-Ras mutated cells into the tumor mass can vary from one patient to another and this fact may substantially influence the ability to predict therapeutic response [26].

Our work includes a large, multi-institutional cohort, involving 263 real-world patients and aims to define the prognostic value of K-Ras mutation rate in a homogeneous population of patient treated with a first-line containing bevacizumab. We decided to consider PFS as the primary endpoint of the study because of its shorter available follow-up time and its independence from additional subsequent therapies, which preserved the homogeneity of our patients' population. Furthermore, some correlation analysis showed associations between PFS and OS in some cancers such as mCRC [27].

We should also admit some limits; first of all the greatest bias of our work derives from the retrospective nature of the study itself. We decided to exclude from the analysis 92 patients because insufficient percentage of tumor cells contained in their samples and other 42 cases because of incomplete follow-up data: this caused a loss of 33% of the original cohort in the final analyses. In addition, the cut-off of 40% (median value) was arbitrarily chosen and not independently validated. Finally despite we used pyrosequencing to assess the mutational status of all patients, the analysis was performed in different laboratory. On the contrary the lack of detection of B-Raf and N-Ras mutations due to their mutually exclusive nature do not represent a limit.

In the first prognostic analysis we confirm the unfavorable K-Ras impact on both survival endpoints; interestingly these results were replicated when we investigated the effect of the mutation rate higher than 40% (chosen as cut-off value) on PFS and OS (PFS; *P* = 0.000; OS; *P* = 0.002), independently of the type of mutation. This strong association emerged in each class of patients analyzed, grouped on the basis of the type of mutation (codon 12, 13 or rare codons), indeed PFS and OS was always higher (in terms of months) in those groups of patients with less than 40% of mutation rate, providing indication of a better survival.

These data encouraged us to apply a multivariate model for the evaluation of the independent effect of mutation rate of codons 12, 13 and rare codons. The analysis' results showed that the prognostic value of exon 2/3 K-Ras mutations is retained after correction for other well-known prognostic factors (age at diagnosis, tumor tissue used for molecular analysis, reference center, number of metastatic sites and first-line chemotherapy) and confirm the robustness of the mutation rate as an effective predictor of PFS and OS.

In conclusion our results demonstrate for the first time a correlation between the K-Ras mutation rate (presence of more or less than 40% mutant alleles) in a homogenous advanced CRC population treated with bevacizumab containing first-line regimens. Certainly, our data need to be confirmed in a validation prospective set, but represent an important proof of concept for future translational analyses.

## MATERIALS AND METHODS

### *In silico* analysis

K-Ras mutation allele frequency was evaluated in Brannon et al cohort consisting of analyzed 69 patient trios of primary CRC, matched metastases and normal tissue retrieved from http://www.cbioportal.org/index.do using the following WEB API command http://www.cbioportal.org/webservice.do?cmd=getMutationData&case_set_id=coadread_mskcc_all&genetic_profile_id=gbm_tcga_mutations&gene_list=KRAS. 20 Kras mutated patients treated with chemotherapy were selected for further analysis. Cut-off estimation for Kaplan Meier analysis for OS was performed through Receiving Operator Curve methods using Cutoff Finder (http://molpath.charite.de/cutoff/).

### Patients and clinical endpoints

This multicentric retrospective study enrolled 397 consecutive patients from 6 Italian cancer centres with a histologically confirmed diagnosis of mutant K-Ras mCRC (evaluation performed homogeneously by pyrosequencing) and treated with first-line anticancer regimens containing bevacizumab. Two clinical endpoints were used: PFS was the time from start of treatment to first evidence of tumor progression or death; OS was the time from start of treatment to death from any cause. The study has been conducted in accordance with the ethical principles that have their origin in the current Declaration of Helsinki and it has been approved by the ethical committee of Campus Bio-Medico of Rome. This study satisfy the REMARK criteria [28].

### Laboratory method

All tumor samples (belonging to primary tumor or metastatic lesions) used in this study were selected and dissected by an experienced pathologist, and were quality controlled by frozen section to ensure that tumor cells were present in at least 60% of the sample. FFPE sections were deparaffinised by submersion in xylene; the tissue was then incubated overnight at 56°C with Proteinase K (Qiagen, UK) to allow samples lysis and DNA was extracted using the Trizol reagent (Invitrogen, CA, USA) following the manufacturer's instructions. To obtain RNA-free genomic DNA, an RNase A (Qiagen, UK) treatment was performed following the protocol instructions. The concentration and purity of the isolated DNA were measured using a NanoDrop ND-1000 Spectrophotometer (Thermo Fisher Scientific, DE, USA). Pyrosequencing analysis was performed by each local laboratory with the exception of cases provide by ASB that were analysed by the coordinating centre. Pyrosequencing analysis of K-Ras status was carried out on 0.15-0.5 pmol of each PCR product using the PyroMark MD System (Qiagen) following the manufacturer's instructions, with sequencing primers and assay parameters specific to each mutation. Codons 12, 13, 61, 117 and 146 of K-Ras gene were analysed. For each assay, pyrosequencing analysis was taken to represent the identified percentage burden of the mutant allele. The cut-off value, discriminating between the mutant and wild-type sequence, was arbitrarily assigned as 10% mutant allele burden. A score for each mutant sequence was used for statistical analysis; in particular we assigned score 0 whether mutant K-Ras alleles were present in less than 40% while score 1 whether mutant K-Ras alleles were present in at least 40%. The determination of 40% as cut off value of allelic frequency was established because it represented the median value of the percentage' distribution of mutant alleles.

### Statistical analysis

PFS was calculated as the period from the date of starting treatment to the first observation of disease progression or to death from any cause. The duration response was defined as the period of time from the initiation of treatment (in a patient responding to therapy) until documentation of radiological or symptomatic disease progression. The OS time was calculated as the period from the date of starting treatment until death from any cause or until the date of the last follow-up, at which point data were censored. PFS and OS were both determined by Kaplan-Meier product-limit method. Finally, the Cox proportional hazards model was applied to the multivariate survival analysis. Survival analysis in multivariate model was corrected for the following variables: age at diagnosis, tumor tissue used for molecular analysis, reference center, number of metastatic sites and chemotherapy regimen in first line. The cut-off point for survival data was May 2014. SPSS software (version 17.00, SPSS, Chicago) was used for statistical analysis. A P value of less than 0.05 was considered to indicate statistical significance.
